# Oligonucleotide surface attachment by tosylation for digital detection of microRNA using photonic resonator absorption microscopy

**DOI:** 10.1063/5.0290900

**Published:** 2025-10-28

**Authors:** Skye Shepherd, Joseph Tibbs, Seemesh Bhaskar, Zayd Bala, Takhmina Ayupova, Lucas D. Akin, Weinan Liu, Marcia H. Monaco, Sharon M. Donovan, Brian T. Cunningham

**Affiliations:** 1 Holonyak Micro and Nanotechnology Laboratory, University of Illinois at Urbana-Champaign, Urbana, Illinois 61801, USA; 2 Department of Bioengineering, University of Illinois at Urbana-Champaign, Urbana, Illinois 61801, USA; 3 Carl R. Woese Institute of Genomic Biology, University of Illinois at Urbana-Champaign, Urbana, Illinois 61801, USA; 4 Department of Electrical and Computer Engineering, University of Illinois at Urbana-Champaign, Urbana, Illinois 61801, USA; 5 Department of Economics, University of Illinois at Urbana-Champaign, Urbana, Illinois 61801, USA; 6 Department of Chemistry, University of Illinois at Urbana-Champaign, Urbana, Illinois 61801, USA; 7 Department of Food Science and Human Nutrition, University of Illinois, Urbana-Champaign, Urbana, Illinois 61801, USA; 8 Division of Nutritional Sciences, University of Illinois, Urbana-Champaign, Urbana, Illinois 61801, USA; 9 Cancer Center at Illinois, University of Illinois at Urbana-Champaign, Urbana, Illinois 61801, USA

## Abstract

DNA capture probes attached to surfaces are necessary for many biosensing assays for the specific detection of nucleic acid target sequences. In this work, we present a chemically mediated direct oligonucleotide–titanium dioxide bond for stable DNA surface immobilization on photonic crystal (PC) biosensors. We validated the DNA–TiO_2_ functionalization using photonic resonator absorption microscopy (PRAM) to digitally detect hybridization-bound nanoparticles, then compared the tosylate-mediated chemical binding method to commonly used silanes (3-aminopropyl)triethoxysilane and (3-glycidyloxypropyl)trimethoxysilane. This surface functionalization process was then applied to the digital detection of nanoparticles for the ultrasensitive detection of microRNA (miRNA) sequences on PRAM. By immobilizing a single-stranded capture DNA sequence onto a titanium dioxide PC surface through a reaction with *p*-toluenesulfonic anhydride, we demonstrate the target recycling amplification process for digital detection of miRNA using PRAM through toehold-mediated strand displacement reactions with linear signal amplification. Using this method of DNA surface functionalization, we achieved attomolar levels of detection of target miRNA-148a-3p, a potential biomarker for certain cancers, without requiring silanes or polyethylene glycol linkers, which can be unstable or expensive. While demonstrated here using PCs for the detection of miRNA, this functionalization approach could be broadly applied to any biosensor with a titanium dioxide surface.

## INTRODUCTION

Surface functionalization and immobilization of nucleic acids is a foundational process in biosensing, which typically involves a specific biorecognition element on a transducer element to detect a target biomolecule.^1,2^ For the detection of nucleic acids through hybridization, the sensitivity, selectivity, and robustness of biosensors depend on consistent and stable high-density surface attachment of DNA capture sequences without crowding.^3–5^ Silane agents such as (3-glycidyloxypropyl)trimethoxysilane (GPTMS) and (3-aminopropyl)triethoxysilane (APTES) have been widely applied to covalently bind DNA sequences or other biomolecules such as antibodies to titanium dioxide surfaces through hydroxylation-activating processes, using vapor or liquid deposition.^6–8^ Silanization methods require precise optimization to create thin, uniform, and stable organosilane layers, with careful attention required for each condition through the steps of hydrolysis, condensation, bond formation, and curing.^9,10^ It is challenging to create stable monolayers that are consistent and reproducible, and silane layers can be affected by hydrolytic instability or various conditions such as humidity and temperature.^11–15^ Additional modifications have been tested in numerous works to chemically modify these silane groups to adjust the surface properties and attach different functionalities, but they typically require exacting reaction conditions and careful optimization.^16,17^ Modifications include the attachment of functionalized polyethylene glycol (PEG) linkers, glutaraldehyde, NHS/EDC coupling, or other alternatives for biomolecule attachment.^7,18–20^ However, efficient and stable attachment of nucleic acids for biosensing remains a challenge.

There are a variety of biosensors that use titanium dioxide for its useful chemical and optical properties. Titanium dioxide has good biocompatibility and non-toxicity, is an excellent photocatalyst, and can be synthesized onto surfaces through inexpensive sputtering or through physical or chemical vapor deposition. Additionally, it is a chemically robust material with a controllable bandgap dependent on crystal phase. This has made it a popular material for photoelectrochemical or amperometric biosensors that use photocatalysis or changes in current for biodetection. TiO_2_ also has a high refractive index and low loss at different optical wavelengths that make it valuable for approaches such as photonic crystals (PCs), optical waveguides, and surface plasmon resonance.

In this work, we demonstrate a new method to attach a nucleic acid capture sequence to a titanium dioxide surface using *p*-toluenesulfonic anhydride and amine-terminated DNA for a photonic crystal (PC) biosensor and digital microRNA (miRNA) detection. This direct attachment approach eliminates the need for PEG or silane linkers, which are typically more costly, may have less accessible chemical headgroups, and can be challenging to optimize for biosensing environments.^21,22^ Tosylate groups, such as those introduced by *p*-toluenesulfonic anhydride on a hydroxylated surface, are a flexible option for surface functionalization due to their potential to be excellent substitution groups for amines or thiols.^23–25^ This has been previously studied for biomolecule conjugation as well as for attachment to other materials such as magnetic beads or cellulose.^26–28^

Our laboratory has previously demonstrated optical biosensing for digital detection using titanium dioxide one-dimensional PCs that act as precise reflectors for specific wavelengths of light. The PCs have been used as optical biosensors for a variety of assays to detect nucleic acids, proteins, nanoparticles, and viruses through silanization with GPTMS or APTES with various linkers for DNA functionalization, antibody attachment, or photonic fluorescent enhancement.^29–33^ PCs can be coupled with optically absorbing nanoparticles that can be digitally counted in images when they are captured on the surface using a red light-emitting diode in a process called photonic resonator absorption microscopy (PRAM) for point-of-care applications.^34,35^ These PCs can also enhance scattering for interferometric scattering microscopy, which resulted in the development of photonic resonator interferometric microscopy (PRISM), a technique that can digitally image individual smaller nanoparticles, viruses, and extracellular vesicles.^36,37^ In this report, we test this new method of DNA attachment using tosylate groups with both PRAM and PRISM for nanoparticle detection and then compare it to previous vapor-deposited GPTMS and a liquid-based APTES functionalization that requires linking chemistry.

**FIG. 1.**
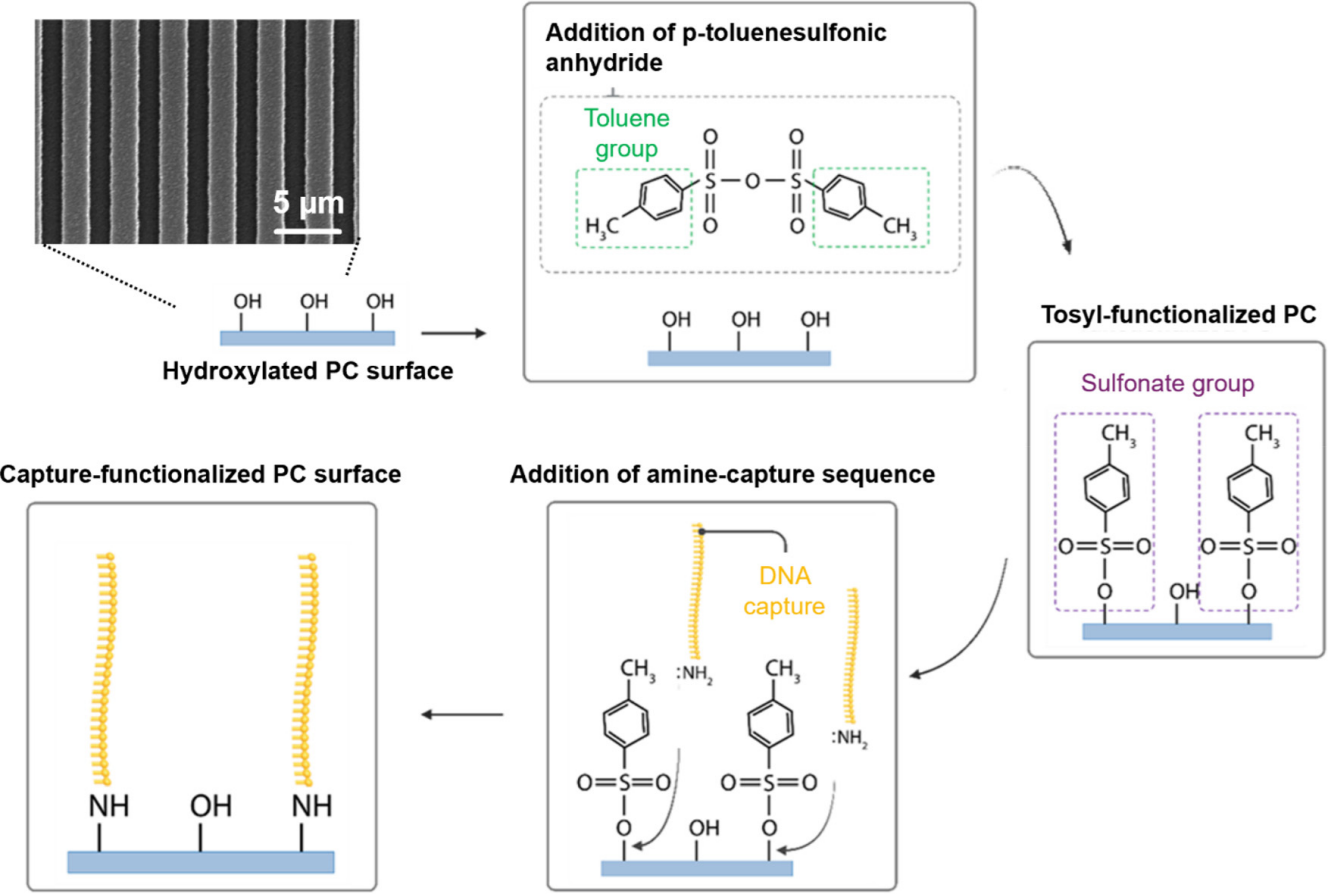
Schematic representation of surface functionalization method for attaching an amine-terminated capture DNA sequence to a titanium dioxide-coated photonic crystal biosensor (SEM image shown) using a tosyl-mediated DNA attachment process.

Recently, a new assay called the target recycling amplification process (TRAP) was developed by Wang *et al.* to amplify the detected signal of microRNA on PRAM using toehold-mediated strand displacement reactions to allow a single target molecule to generate multiple PC-attached gold nanoparticles. This assay was applied with PRAM to detect miR-375 and miR-21 from exosomal microRNA.^38^ However, the TRAP assay previously relied on GPTMS-silanized PCs for DNA capture immobilization, with a protocol that requires a glove bag, long silanization times in a vacuum oven, and is very sensitive to humidity or temperature changes. In this work, our approach instead leverages the *p*-toluenesulfonic anhydride-mediated method of chemical nucleic acid attachment, shown in Fig. 1, which provides a more stable bond for DNA on the PC TiO_2_ surface while avoiding the limitations of silanes. We then demonstrated this capture approach by designing and applying TRAP probes for a new microRNA target, miR-148a-3p, which is a biomarker of interest in angiogenesis, breast cancer, and prostate cancer.^39–41^ Using a newly developed portable version of the PRAM instrument,^42,43^ we demonstrated attomolar levels of detection for miR-148a-3p using TRAP with the tosylate surface chemistry, showing potential for faster nucleic acid detection and broader point-of-care applications. Our findings demonstrate that this direct DNA–TiO_2_ attachment strategy is well-suited for high-sensitivity nucleic acid detection on other photonic biosensing platforms that also utilize TiO_2_ surfaces, making it a valuable technique for biosensing applications that require robust, low-cost DNA immobilization.

## RESULTS AND DISCUSSION

### Surface characterization of *p*-toluenesulfonic anhydride or alkoxysilanes on titanium dioxide surfaces

**FIG. 2.**
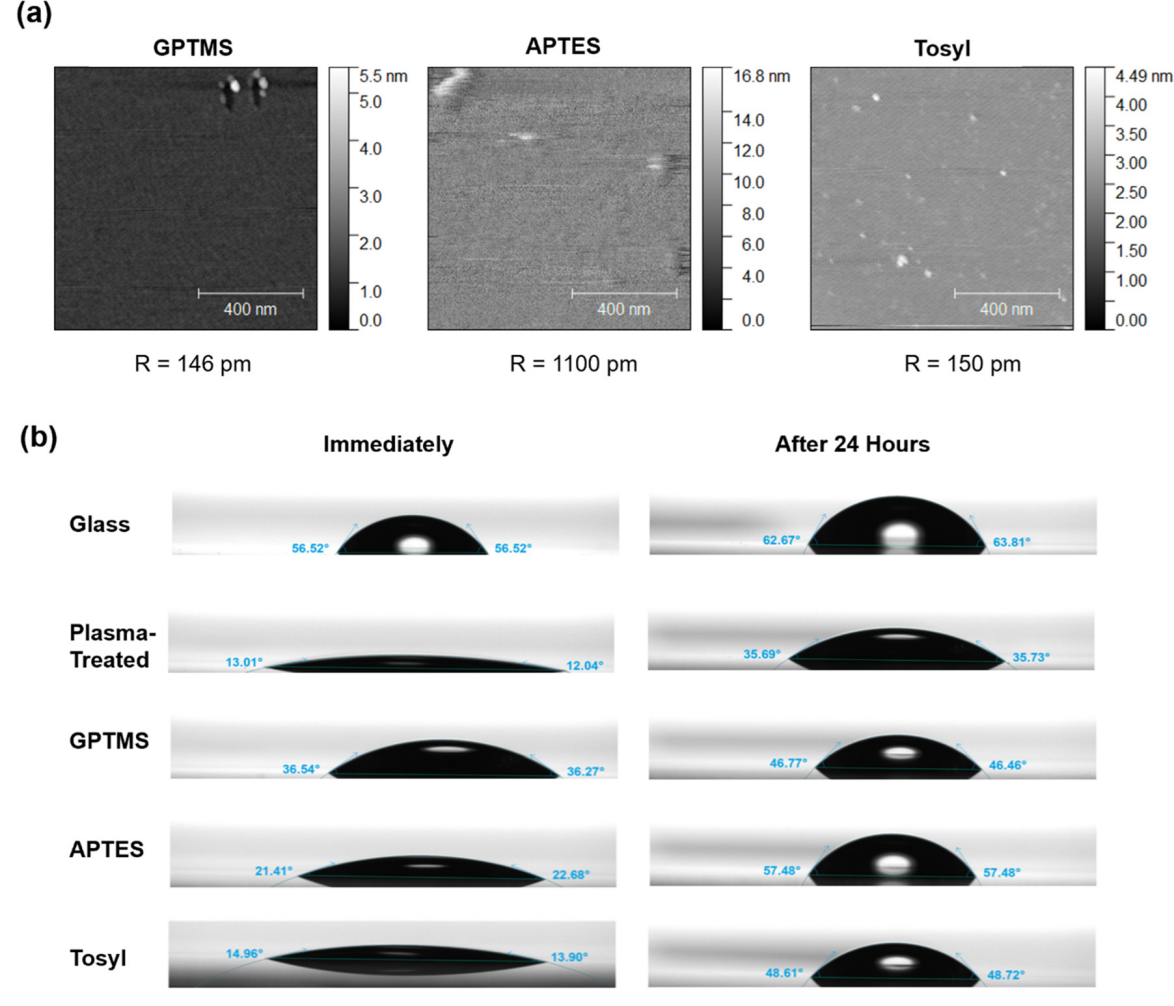
Surface characterization of different functionalization methods. (a) Atomic force microscopy for mica functionalized by either vapor-deposited GPTMS, solution-based APTES, or solution-based tosyl, with calculated surface roughness. (b) Comparison of contact angle on functionalized glass surfaces on glass, plasma-treated glass, or using either vapor-deposited GPTMS, solution-based APTES, or solution-based tosyl. Contact angle was measured immediately after functionalization or after storage in a vacuum for 24 h.

To initially characterize the tosyl functionalization process, flat planar mica was functionalized with either a vapor-deposited GPTMS or solution-based APTES, shown in Supplementary Fig. S1, or with the solution-based *p*-toluenesulfonic anhydride (Fig. 1). Vapor silanization used a vacuum oven at 80 °C and −25 Torr for 6 h, with 100 *μ*l of GPTMS to deposit thin layers of silane, while solution-based methods used either 2% APTES in 95% ethanol and 5% water or 5 mg of *p*-toluenesulfonic anhydride dissolved in 50 ml of anhydrous toluene, which were both reacted for 1 h at room temperature. All three methods are described in detail in the section on Methods. The three surface functionalization methods were compared with atomic force microscopy (AFM) [Fig. 2(a)] on mica, where the tosyl liquid-functionalized method showed similar roughness values to the GPTMS vapor-functionalization, which was both near the planar roughness of mica. The tosyl sample displayed a few small islands of ∼5 nm in height scattered across the surface, while the GPTMS sample had some larger aggregates. They both were smoother than the solution-based silanization method using APTES, which created significant surface roughness and some infrequent islands over 15 nm in height. Reduced surface roughness is indicative that the chemical functionalization is forming fewer multilayers, as opposed to aggregates that may induce unwanted chemical moieties. Smoother surfaces and thin surface functionalization layers also maintain the characteristic optical properties of the surface. Finally, rough surfaces and islands of aggregates can create high-energy sites that are prone to nonspecific binding. The three surface functionalization methods were also examined by AFM on the titanium dioxide PC biosensor surfaces, according to methods used previously in Bhaskar *et al.*, along with an untreated control PC, which is shown in Supplementary Fig. S2.^44^ Similarly, the tosyl-treated PC displayed less surface roughness than the other functionalization options. This demonstrates the potential of tosyl-functionalized surfaces for reduced surface roughness, without requiring vacuum ovens or complicated methods. Contact angle and wettability of functionalized surfaces were also characterized by a tensiometer on glass [Fig. 2(b)]. GPTMS, APTES, and tosylated glass surfaces were tested, along with bare glass cover slides and samples that were plasma treated. Each surface was tested immediately after treatment and after storage in a vacuum desiccator for 24 h. The functionalization methods all created a surface that resulted in higher wettability than glass but less hydrophilic behavior than plasma treatment. All methods demonstrated increased contact angle after storage, likely due to the time since oxygen-plasma treatment.

### Optimization of DNA attachment to PCs using the tosylate reaction

**FIG. 3.**
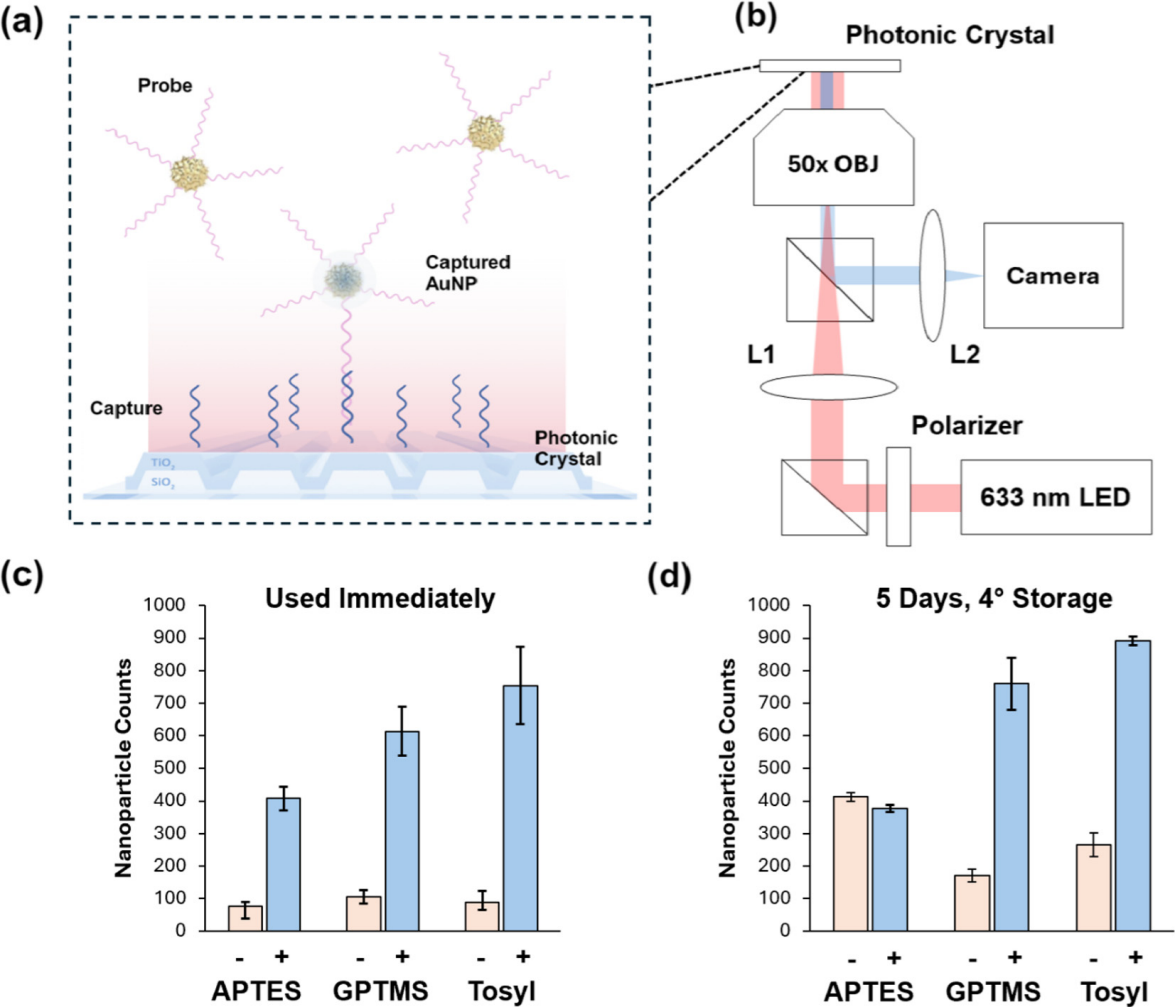
Application of different functionalization methods using PRAM. (a) PCs functionalized with single-stranded capture DNA allow AuNPs captured through hybridization to probe DNA to be counted. (b) Portable PRAM layout and light path, using a 633 nm red LED, beam splitters, and lenses to direct light through an objective that reflects off the PC back to a CMOS camera. (c) Direct AuNP probe hybridization to capture DNA on PC comparison assay to compare GPTMS, APTES, and tosyl approaches for surface functionalization of capture sequence, with a non-complementary probe sequence used as a control. Standard deviation is shown (n = 3 trials). (d) Testing of PCs 5 days after functionalization and DNA addition, stored at 4 °C. Standard deviation is shown (n = 3 trials).

The tosyl-functionalized PCs were reacted with amine-terminated DNA to immobilize capture sequences on the titanium dioxide surface, as shown in Fig. 1. Gold 80 nm diameter nanoUrchins (AuNPs), which are selected to highly absorb wavelengths near 620 nm [Fig. S3(a)] due to localized surface plasmon resonance, are then functionalized with a complementary probe DNA sequence that can hybridize to the immobilized capture sequence. When these nanoparticles are bound to the red-tuned PCs, which resonantly reflect red light with high efficiency [Figs. S3(b) and S3(c)], they can be imaged by the photonic resonator absorption microscope (PRAM). The PRAM system captures the reflected light of a red-emitting LED centered at 633 nm, which is strongly absorbed when a nanoparticle binds to the PC [Fig. 3(a)], causing a localized decrease in reflected light intensity observed by an image sensor. Each dark location represents the presence of one surface-captured AuNP that can be digitally counted [Fig. 3(b)] by image processing (Fig. S4). Using the PRAM, the probe-AuNPs (pink sequences) were used to compare the three different surface functionalization methods to examine the amine-terminated capture DNA (blue sequences) density and stability across the PC surface. The APTES method used an additional disuccinimidyl carbonate (DSC) linker step to create an amine-active surface, while the GPTMS and tosyl methods were directly reacted with the capture DNA. Each surface functionalization method was effective at generating a high signal-to-noise ratio between a positive nanoparticle signal of probe-AuNPs and a negative control with AuNPs bound to a non-complementary probe, as shown in Fig. 3(c). This indicates that GPTMS, APTES, and the tosyl method are all successful at attaching an amine-terminated nucleic acid to titanium dioxide surfaces. When the PCs were functionalized and then stored for 5 days at 4 °C with the immobilized capture DNA in solution, as shown in Fig. 3(d), the tosyl-immobilized DNA showed greater stability than the GPTMS and APTES methods, which displayed reduced signal response when tested on PRAM. The tosyl PC still showed higher reactivity, indicating that the DNA–titanium dioxide bond was stable and did not degrade in water.

Additionally, the new method of surface functionalization was examined using the photonic resonator interferometric scattering microscope (PRISM) to compare the GPTMS functionalization and tosyl. The PRISM system can digitally count smaller nanoparticles, and PRISM has a much smaller field of view (FOV) (30 *μ*m by 30 *μ*m) compared to the PRAM system (380 × 240 *μ*m^2^). This allows closer observation of the density of binding sites across the surface. To determine the uniformity of surface attachment at a smaller scale, the PRISM instrument equipped with a 100× objective lens was used to capture images with a 30 *μ*m × 30 *μ*m field of view.^36^ At this length scale, variations in functionalization density for all methods became significant, and FOVs demonstrated more variation in particle counts than would be expected from Poisson statistics alone. The best explanation for this behavior is the plasma treatment by the barrel asher (PicoDiener) could introduce variations in surface activation that may be ameliorated using a capacitively coupled oxygen activation system or a reactive ion etch (RIE) system. The two methods were tested in different conditions and buffers using 30 nm diameter gold nanoparticles that can be imaged through their localized scattering of laser illumination (Figs. S6 and S7). The tosyl method demonstrated comparable levels of nanoparticle binding for lower levels of DNA, suggesting good binding of immobilized capture DNA, but saturated at lower total nanoparticle levels per FOV (Fig. S7). This indicates that the liquid-based tosyl method still has potentially fewer reaction sites than the vapor-based method of GPTMS silane deposition but can still be effective at applications that do not require extremely high-density capture molecules. The GPTMS method had an average of 117 particles/FOV, while the tosylate reaction had an average of 34.7 particles/FOV. For the 30 *μ*m by *μ*m PRISM FOV, this means that GPTMS and tosyl can capture around 0.13 and 0.0385 particles/*μ*m^2^, respectively. The tosylate method demonstrates a lower estimated surface density than the GPTMS approach, but with a larger field-of-view in the PRAM system (250 × 350 *μ*m), each nanoparticle image takes an estimated 1 × 1 *μ*m of space. This means that for this large-FOV digital application with PRAM, high-density capture is not necessary, as each 80 nm diameter nanoparticle counted will be highly separated from its digitally counted neighbors. The GPTMS functionalization demonstrated a higher capture density overall, which is important for systems that image only a small FOV. However, for applications where a larger area is scanned, such as PRAM, both counting statistics and overall capture density are less important.

### Detection of miR-148a-3p using the target recycling amplification process (TRAP) and tosylate-immobilized capture DNA on PRAM

**FIG. 4.**
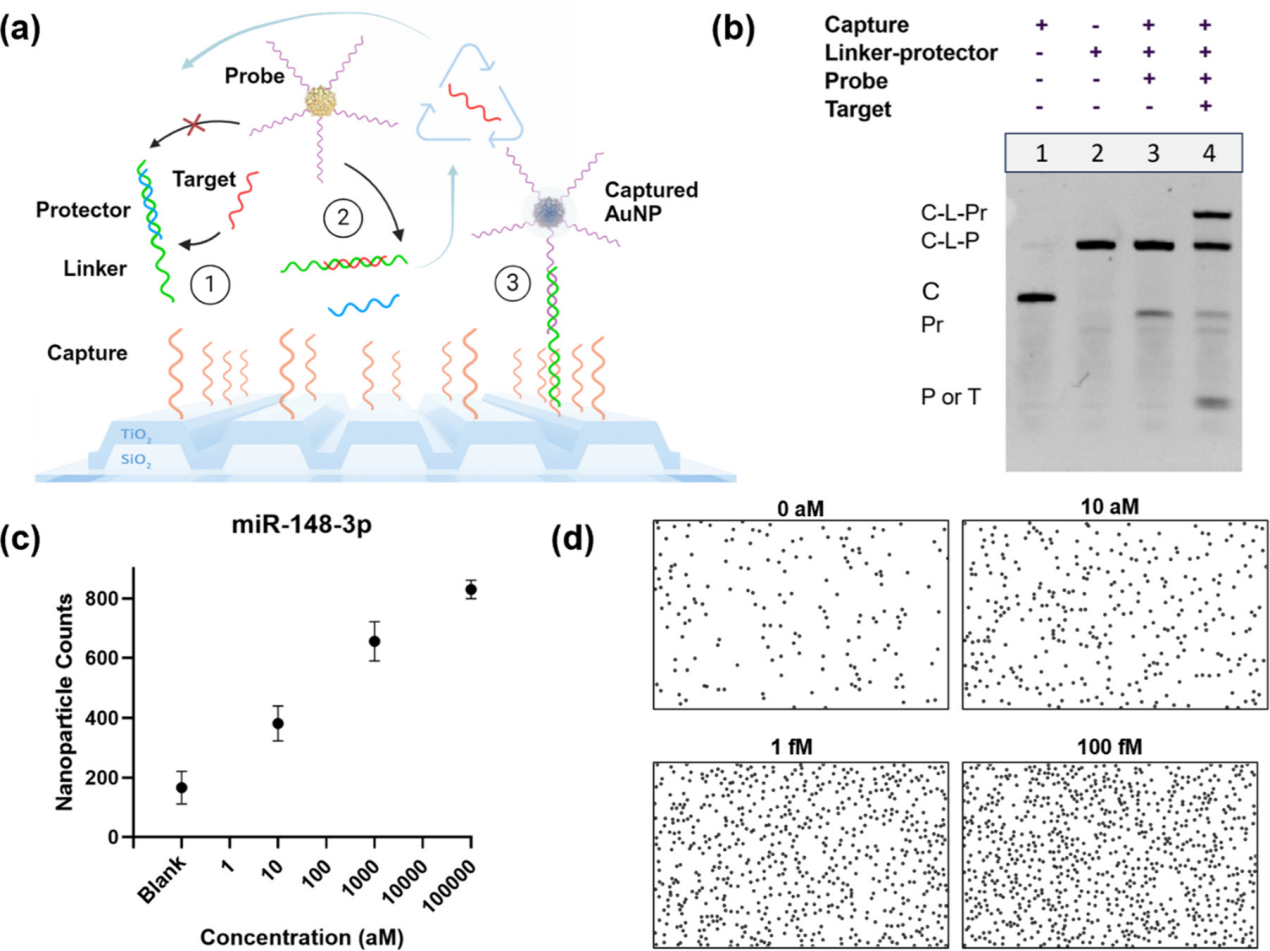
Detection of microRNA using tosylate-immobilized capture DNA with the target recycling amplification process (TRAP). (a) Process of nucleic acid toehold-mediated strand displacement for signal amplification of digitally detected nanoparticles. (b) Native PAGE testing of DNA probes for TRAP nucleic acid amplification of miR-148a. (c) Standard curve for detection of miR-148a-3p using TRAP on PRAM with tosylate-immobilized capture DNA, with images taken at 30 min. Standard error shown (for n = 3 trials). (d) PRAM images of nanoparticles for different concentrations of miR-148a-3p for a representative trial detected by TRAP, also at 30 min.

To demonstrate the tosyl-immobilized testing for biosensing, we applied a toehold-mediated strand displacement reaction circuit called the target recycling amplification process (TRAP) to amplify the microRNA signal before digital detection with the PRAM instrument. In the TRAP assay, shown in Fig. 4(a), the target microRNA (red) can bind to an initial toehold on the linker substrate (green), displacing the protector strand (blue). This exposes a second toehold on the linker strand, which reacts with the probe sequence (purple), binding a nanoparticle. The reaction with the probe simultaneously displaces the target microRNA back into the solution, allowing a single microRNA to bind multiple nanoparticles, thus linearly amplifying the generated immobilized AuNP count for detection. Sequences for TRAP were developed and optimized for a new microRNA target, miR-148-3p, which was designed in NUPACK to limit cross-reactivity and secondary structures and to examine the thermodynamics of the toehold sequences. The optimized sequences were then tested in a native polyacrylamide gel electrophoresis to examine amplification [Fig. 4(b)]. In the negative control (lane 3), the probe is unable to displace the protector, as the toehold is sequestered. When the target miR-148-3p sequence is added in the positive control (lane 4), it displaces the protector sequence, allowing the probe to react and form the final triplex of capture, linker, and probe that can result in a PC-attached AuNP that can be detected by PRAM.

The TRAP sequences were then tested using the tosyl-immobilized capture DNA on PRAM with TRAP-functionalized AuNPs to detect synthetic miR-148-3p. Three concentrations were tested at 10 aM, 1 fM, and 100 fM, along with a blank negative control that did not have added microRNA [Fig. 4(c)]. Each concentration was repeated in triplicate experiments on different PCs. Higher concentrations were not shown as the surface of countable AuNPs saturates. The tosyl-TRAP assay demonstrated attomolar-level detection of miR-148-3p, with a calculated LOD of 2.4 aM in buffer (Fig. S8), which was similar to previous results with other microRNAs previously tested with TRAP. Representative images from the PRAM assay for each concentration after image processing and digital counting are shown in Fig. 4(d). TRAP is not run to a complete end point, to minimize nonspecific binding and nanoparticle settling, and the assay will continue to react until imaging. The assay acts as an entropy-driven toehold exchange reaction, where each toehold has a reverse reaction as well as a forward reaction, so increasingly high levels of target miRNA will cause the target-to-probe displacement reaction to slow. The TRAP assay was also tested using GPTMS, shown in Fig. S9, which had comparable results, demonstrating a limit of detection of 7.8 aM for miR-148. The tosyl-TRAP approach was finally established in spiked plasma samples (Fig. S10), which demonstrated comparable results to the assay run in buffer. This establishes the potential for this method for future testing of microRNA in clinical samples. This approach has been compared to several other standard and novel methods of microRNA detection in Table S3.

This demonstrates the ability to achieve rapid and sensitive detection using this method of surface functionalization for DNA-based titanium dioxide sensors. This also represents a potential low-cost method for DNA attachment to photonic crystal surfaces. When considered at per-PC functionalization, the GPTMS and APTES-DSC approaches cost approximately $0.17 and $0.26, respectively, while the tosyl approach costs only $0.06 per PC. However, the GPTMS method also additionally requires an expensive vacuum oven, while the tosyl and APTES methods can be performed at room temperature and in a normal atmosphere. Reagent costs and calculations are shown in Supplementary Table S2.

## CONCLUSION

In conclusion, in this work, we describe a new method to functionalize photonic crystal biosensors with nucleic acid probes for digital biosensing, offering a stable alternative to conventional silane chemistry. Three functionalization protocols were carried out using a gas-phase GPTMS, liquid-based APTES, and a linker, or *p*-toluenesulfonic anhydride, to attach amine-terminated DNA capture sequences to the titanium dioxide surfaces of PCs. These methods were examined by atomic force microscopy for surface roughness, and we characterized the effect on wettability and contact angle. DNA immobilization for capture sequences was also compared using both photonic resonator absorption microscopy and photonic resonator interferometric scattering microscopy to test hybridization of complementary and non-complementary probe-functionalized nanoparticles, confirming effective DNA attachment. Finally, the new tosyl method was applied for the ultrasensitive detection of the new target miR-148-3p, a cancer-associated biomarker, using the target recycling amplification process to non-enzymatically generate higher signals before digital detection. This demonstrates the use of tosyl for DNA attachment for ultrasensitive optical biosensing, especially for point-of-care applications.

## METHODS

### Nucleic acids

DNA sequences were purchased from IDT (Coralville, IA), with standard desalting. All sequences are listed in Table S1. Capture or probe sequences for conjugation included a 15-nucleotide poly T spacer. Capture sequences had amine terminal groups. Sequences for nanoparticle functionalization were thiol-terminated. Nucleic acids were stored at 4 °C for short-term use or −20 °C for long-term storage.

### Preparation of self-assembled thiol-gold AuNP–ssDNA conjugates (Pr-AuNPs)

Conjugation of thiolated-DNA sequences using self-assembly to bare 80 nm gold nanoUrchins (Cytodiagnostics SKU: GU-80-20). Thiolated 100 *μ*M DNA probes were reduced using 100 equivalents of TCEP or DTT, then purified using 0.5 ml 3 K Amicon centrifuge filters by centrifugation at 14.0 rcf for 15 min, three times. Reduced probe sequences were immediately used or frozen at −20 °C until use. 20 *μ*l of 5 *μ*M reduced and purified thiolated probe sequences were added to 1 ml of 1 OD of nanoparticles, at a concentration of ${7.82\times 10}^{9}$ particles/ml. The probes were allowed to react for 15 min at room temperature, then 2 *μ*l of 4 mg/ml of a 1 K mPEG-SH (Creative PEGWorks) was added to additionally block the gold surface from nonspecific binding. Both the probe and PEG were allowed to assemble on the gold surface for 48 h at room temperature on a shaker at 400 rpm to prevent settling, then excess unbound probe and PEG complexes were removed by centrifuging three times at 600 rcf for 30 min and washing with a 1× TE, 0.025% tween20 buffer. AuNPs were stored at 4 °C for up to 2 weeks until use.

### Pre-functionalization photonic crystal surface preparation

Photonic crystals were glued to cover slips using UV-curing adhesives (Norland Optical Adhesive 63). PCs were then cleaned through sonication in acetone, IPA, and MilliQ® water for 2 min each. PCs were dried by heating to 120 °C for 5 min, then oxygen plasma treated using a PicoDiener System at 100% power, 0.8 mbar for 10 min. PCs were then immediately used for either tosylate, GPTMS, or APTES surface functionalization for DNA attachment.

### *p*-toluenesulfonic anhydride surface functionalization and capture DNA attachment

5 mg of *p*-toluenesulfonic anhydride was dissolved in 50 ml of anhydrous toluene in a glass staining jar in a nitrogen-filled glove bag. Up to four oxygen plasma-treated PCs were added for 1 h at room temperature, then removed and washed with toluene, then water for 2 min by sonication. 50 *μ*M of amine-terminated capture DNA in water was incubated overnight at room temperature, then excess capture was removed by washing five times using 1× TE, 0.025% tween20. PC surfaces were blocked for 30 min at room temperature using PBS Super-Block (Thermo Fisher Scientific, #37580) blocking buffer, then PCs were washed once before use.

### GPTMS vapor-based surface functionalization and capture DNA attachment

250 *μ*l of (3-glycidyloxypropyl)trimethoxysilane (GPTMS) was added into the bottom of horizontal glass staining jars in a nitrogen-filled glove bag. An oxygen plasma-treated PC was suspended face down above the silane on glass slides. Jars were placed in an 80 °C vacuum oven at −25 Torr for 6 h, then were removed and washed with toluene, methanol, and water for 2 min each by sonication. 50 *μ*M of amine-terminated capture DNA in 1× PBS, 8.5 pH was incubated overnight at room temperature, then excess capture was removed by washing five times in 1× TE, 0.025% tween20. PC surfaces were blocked for 30 min at room temperature using PBS Super-Block, then washed once before use.

### APTES solution-based surface functionalization and capture DNA attachment

2% (3-Aminopropyl)triethoxysilane (APTES, 99% purity, Sigma-Aldrich, #440140) in 95% ethanol to 5% water was added to a 50 ml (49 ml solvent and 1 ml APTES) glass staining jar in a nitrogen-filled glove bag. Up to four oxygen plasma-treated PCs were added to react with APTES solution for 1 h at room temperature, then the PCs were rinsed in acetone, ethanol, and water for 2 min each by sonication. Disuccinimidyl carbonate (DSC) was added at 10 mM in 10% DMSO solution and allowed to react for 20 min at room temperature, then was washed using 1% DMSO, then water. 50 *μ*M of amine-terminated capture DNA solution was added for 3 h at room temperature, then excess unbound capture was removed by washing with 1× TE, 0.025% tween20 five times. PC surfaces were blocked for 30 min at room temperature using PBS Super-Block, then washed once before use.

### Direct nanoparticle-PC hybridization on PRAM

To directly compare different surface functionalization methods, single-stranded DNA probe-functionalized AuNPs were tested with a complementary capture sequence attached using the three surface functionalization methods. A background reference group was also tested for each with AuNPs functionalized with a non-complementary DNA sequence. Functionalized PCs with attached 20 *μ*l PDMS-formed wells then had thiol-conjugated probe-AuNPs added at a final concentration of ${3.4\times 10}^{9}$ particles/ml. The AuNPs were allowed to bind for 20 min, then were imaged using the photonic resonator absorption microscope. The negative control for background AuNPs used a non-complementary thiolated probe sequence (NC-Pr in Table S1) that would not hybridize with the capture sequence. To test storage, the same experiment was repeated, but functionalized PCs were stored at 4 °C with capture DNA solution, then washed with 1× TE and 0.025% tween20 five times. PC surfaces were blocked for 30 min at room temperature using PBS Super-Block, then washed once before use.

### Duplex annealing

Duplex linker-148 and protector-148 sequences for TRAP assays were annealed in a buffer of 1× Tris-EDTA, 12.5 mM MgCl_2_ at a pH of 7.4 by mixing in a 1:2 stoichiometric ratio—with concentrations of 10 *μ*M linker and 20 *μ*M protector, with the protector strands in excess. The solution of linker–protectors was annealed by then heating to 90 °C and slowly cooling to room temperature. Annealed duplexes were then stored at 4 °C for short-term use or −20 °C for long-term storage.

### NUPACK sequence design for TRAP detection of miR-148

Custom-designed nucleic acid sequences used in the TRAP assay were created according to previous work in Wang *et al.*,^38^ where the first toehold region consists of five nucleotides, and the second toehold region has seven nucleotides, with a two-nucleotide initial inactive overhang. Sequences were examined in NUPACK55 for the presence of competing secondary structures, cross-reactivity, or thermodynamic changes. Sequences were then tested empirically using off-surface TRAP assays, which were visualized by native polyacrylamide gel electrophoresis to examine binding and recycling, or potential off-target activity.

### Polyacrylamide gel electrophoresis testing of miR-148-3p amplification

Sequences were tested at a standard concentration of 100 nM, all in stoichiometrically equal concentrations, except for the target miR-148-3p sequence, which was prepared at a tenfold lower concentration of 10 nM. After reacting for 2 h at room temperature in a 1× TE, 5 mM MgCl_2_ buffer (pH of 7.4), sequences were separated by electrophoresis in a 12% polyacrylamide native gel run at 155 V for 35 min, stained with GelRed (Gold Bio), and imaged using a GelDoc XR (BioRad) with trans UV illumination. Lanes tested capture–linker–probe hybridization with and without added miR-148 target for a positive and negative control of amplification design.

### TRAP detection assay for microRNA-148a-3p

Pre-annealed linker–protector duplexes (20 pM) at a ratio of 1:2 linker to protector, functionalized nanoparticles (final OD of 0.5), and a spike-in microRNA-148a target at three concentrations (10 aM, 1 fM, and 100 fM), along with a negative control, were added to a *p*-toluenesulfonic DNA-functionalized surface after Super-Block was removed in PDMS wells in a 1× TE, 5 mM MgCl buffer. Each concentration was reacted for 30 min on the PC surface, then imaged using the portable PRAM system. Counts from each FOV were averaged. Data were repeated in separate triplicate experiments to establish the miR-148a standard curve. For the plasma tests, miR-148 was spiked into human plasma (pooled human plasma, K2 ETDA, Innovative Research, #50-203-6374), which was then tested at 0 aM, 10 aM, 1 fM, and 100 fM, where human plasma was one-tenth of the final reaction volume.

### Scanning electron microscopy (SEM)

The photonic crystal was sputtered with a thin layer (2 nm) of gold palladium (Au/Pd). The SEM images were collected using a Field-Emission Environmental Scanning Electron Microscope (FEI Company) at 50 000× magnification at a 30 kV beam intensity.

### Atomic force microscopy (AFM)

Atomic force microscopy images and surface roughness measurements were collected using an NX20 atomic force microscope (Park Systems) on freshly cleaved planar muscovite mica surfaces (Sigma-Aldrich, AFM-71855-15-10), which were oxygen plasma treated and then functionalized with either *p*-toluensulfonic anhydride, APTES, or GPTMS alkoxysilanes. All AFM data were collected in “tapping” mode. Images were also taken for various surface chemistry functionalizations on photonic crystals, shown in Fig. S6.

### Contact angle measurements

Surface contact angle measurements were collected using a Theta Lite optical tensiometer (BioLin Scientific). Glass slides were prepared using the previously described functionalization methods in this paper; untreated coverslips and those subjected to 10 min of oxygen plasma were also measured for comparison. All slides were stored in MilliQ water for 30 min before being rinsed in IPA and dried under a stream of nitrogen. Then, the tensiometer was used to dispense 5 *μ*l of water onto the surface in parallel with active video recording at an acquisition rate of 33 frames per second (fps). Image analysis was accomplished using the OneAttension software package provided with the instrument (BioLin Scientific), and contact angle was approximated using the Young–Laplace model of surface tension. The left and right contact angles were averaged to determine the approximate contact angle of each surface.

### PRISM comparison of GPTMS and *p*-toluenesulfonic anhydride DNA immobilization

30 nm AuNPs were prepared with a functionalization of a capture strand complementary to the tether strand. PRISM-compatible photonic crystal substrates were functionalized using the gas-phase GPTMS deposition (6 h in a vacuum oven at 80 °C) or the p-TSA method described in the main text. Aqueous solutions of 10, 25, or 50 *μ*M amine-terminated capture strands suspended in 50 mM borate buffer (pH 8.5) were supplied to the surfaces in 3 mm diameter PDMS wells for a final volume of 20 *μ*l per well. After overnight incubation at 4 °C, excess capture strands were removed from the PC surface by sequential buffer rinse cycles. After rinsing, 40 *μ*l of freshly prepared suspension of 30 nm gold nanoparticles at 1010 nanoparticles/ml were added to the surface. PRISM imaging was done on the device described previously, collecting 10 fields of view (30 × 30 *μ*m) for each experimental condition.

### Nanoparticle digital counting

Starting with the raw image captured by PRAM, we first applied a Tophat filter and Fourier DC component removal algorithm to eliminate non-uniform background. The noise of the image was suppressed through a simple Wiener filter. Next, we applied the maximally stable extremal regions (MSER) extremum searching algorithm to recognize individual surface-proximal AuNPs, using features including the threshold of gray scale, size, relative circularity, Euler number, and equivalent diameters as criteria to seek the extreme regions generated from the AuNPs. To clearly visualize the particle counting process, the detected AuNPs were marked with identical circles on a blank background at their corresponding centroid location, and the total number detected was reported in the final count.

## SUPPLEMENTARY MATERIAL

See the supplementary material for the materials, characterization experiments of AFM, PRISM experiments, nucleic acid sequences (Table S1), microscope setup, and reagent costs (Table S2). Structures of GPTMS and APTES on PC (Fig. S1), AFM for PC data (Fig. S2), nanoparticle and PC spectra characteristics (Fig. S3), raw and counted PRAM images (Fig. S4), PRISM layout (Fig. S5), raw and processed PRISM images (Fig. S6), GPTMS and tosyl surface density comparison (Fig. S7), miR-148-3p LOD tosyl standard curve (Fig. S8), miR-148-3p LOD GPTMS standard curve (Fig. S9), comparison in buffer and plasma for miR-148 detection using tosyl (Fig. S10), and a table comparing microRNA detection methods (Table S3).

## Data Availability

The data that support the findings of this study are available within the article and its supplementary material.
